# Gait training using a robotic hip exoskeleton improves metabolic gait efficiency in the elderly

**DOI:** 10.1038/s41598-019-43628-2

**Published:** 2019-05-09

**Authors:** Elena Martini, Simona Crea, Andrea Parri, Luca Bastiani, Ugo Faraguna, Zach McKinney, Raffaello Molino-Lova, Lorenza Pratali, Nicola Vitiello

**Affiliations:** 1 The BioRobotics Institute, Scuola Superiore Sant’Anna, Pisa, Italy; 2 Fondazione Don Carlo Gnocchi, Milan, Italy; 3 Institute of Clinical Physiology, National Research Council, Pisa, Italy

**Keywords:** Geriatrics, Biomedical engineering

## Abstract

Robotic exoskeletons are regarded as promising technologies for neurological gait rehabilitation but have been investigated comparatively little as training aides to facilitate active aging in the elderly. This study investigated the feasibility of an exoskeletal Active Pelvis Orthosis (APO) for cardiopulmonary gait training in the elderly. Ten healthy elderly volunteers exhibited a decreased (−26.6 ± 16.1%) Metabolic Cost of Transport (MCoT) during treadmill walking following a 4-week APO-assisted training program, while no significant changes were observed for a randomly assigned control group (n = 10) performing traditional self-paced overground walking. Moreover, robot-assisted locomotion was found to require 4.24 ± 2.57% less oxygen consumption than free treadmill walking at the same speed. These findings support the adoption of exoskeletal devices for the training of frail individuals, thus opening new possibilities for sustainable strategies for healthy aging.

## Introduction

With the present aging of the worldwide population, the problem of age-related mobility loss poses serious social implications^1,2^. In fact, the decline of physical function with age ultimately leads to sedentary lifestyles, which are strongly correlated with various cardiovascular and metabolic morbidities and reduced life expectancies^3–5^. Such implications are heavily taxing the sustainability of global healthcare systems and welfare policies, while reducing individual quality of life. As a result, the concept of “active-” or “successful aging” has emerged in recent years as a strategy to promote physical and mental health through aging, primarily by the adoption of active individual behaviors^2,6,7^.

Regular physical activity is recommended as a primary countermeasure to mitigate the natural decline of physical function with age. However, the limited mobility of many older adults constitutes a formidable barrier to the regular practice of standard exercise programs. Indeed, the elderly face higher metabolic demands for activities of daily living^8,9^, exhibit slower walking speeds and limited ranges of motion^8,10^, and commonly suffer from lower-limb pain and balance disorders^11,12^, thus exposing them to increased risk of falling^13,14^. Moreover, various comorbidities and diminished psychological health, together with other environmental challenges, can further undermine the regular practice of physical exercise. Hence, specific training paradigms accounting for age-related frailty and disabilities must be designed for the elderly^15–18^.

Walking represents an advantageous choice of exercise for older adults: a moderate-intensity activity such as brisk walking has been found to yield important benefits to cardiovascular health, while remaining an accessible activity for long-term adherence^19^. In addition to aerobic exercise, strength training has been recommended for the elderly as a preventive strategy for reducing fall risk^17–20^. In this vein, there has been increasing interest in eccentric exercise strategies, based on the higher force generation capacity of eccentric relative to concentric contractions, as well as the increased oxygen economy of eccentric contractions for equivalent-force outputs^21^. Such high efficiency, together with growing evidence that aging does not severely affect eccentric strength, makes training strategies based on negative muscular work potentially ideal for the training and rehabilitation of frail populations such as the elderly^21–23^. Several studies have demonstrated the superiority of eccentric strength training over the traditional approach in increasing muscular strength and functional abilities of older adults^24,25^.

In general, exercise prescriptions for individuals with limited mobility require careful planning. Common guidelines recommend low initial intensities, avoidance of high-impact activities, and individually tailored plans^18,26^. Within this framework, technological innovation can provide valuable tools that promote user engagement and allow for controlled, gradual progression of training intensities^27,28^.

Exoskeletal robotics, for example, have emerged as a promising class of technology to either preserve or restore mobility for health and independence^29–31^. With neurologic patients, robot-assisted rehabilitation programs have been shown to improve walking efficiency of stroke survivors^32–34^, and to restore ambulatory capabilities in spinal cord injury (SCI) patients with complete lesions^35,36^. By comparison, fewer studies have focused on the effects of exoskeletons in neurologically intact elderly users, and such studies have predominantly pursued the reduction of locomotion energy expenditure during short-term, assistive use of the devices, rather than long-term training for improved efficiency of unassisted gait^37–39^.

As a further application, exoskeletons may have the potential to elicit “pre-habilitative” effects in the elderly when used as training tools during regular physical activity. In this regard, exoskeletons may be more effective than traditional exercise, by enabling optimized training paradigms. For example, they may enable training at higher velocities, volumes, or workloads without significantly increasing the physical effort required to the user. They may also be used to train novel movements and refine muscular coordination under safely controlled conditions. Such uses of exoskeletons would likely make exercise prescriptions for people with reduced mobility more amenable and sustainable for long-term daily practice.

With respect to the clinical objectives and quantifiable outcomes of active aging strategies, low functional independence in the elderly has been attributed in large part to reduced gait efficiency^40^. Thus, the Metabolic Cost of Transport (MCoT) — i.e. the energy expenditure per unit distance walked — can serve as a good multi-purpose health metric for the effectiveness of active aging strategies and interventions.

In this study, we tested the effectiveness of a 4-week robot-assisted gait training program in decreasing MCoT in 10 healthy elderly subjects, relative to a randomized control group (n = 10) performing a traditional home exercise program of self-paced overground walking. The adopted training protocol employed an integrated training strategy, combining aerobic and strength training elements in a brisk treadmill walking exercise. Here, a powered hip exoskeleton called the Active Pelvis Orthosis (APO) provided a gait phase-modulated torque profile specifically designed to enhance eccentric activation of hip extensors while providing hip flexor assistance during swing, to reduce the total energetic cost of walking. This combined resistance-assistance profile sought to capitalize on recent findings on the effectiveness of eccentric strength training for the elderly while adhering to acknowledged clinical recommendations of sustainable long-term activity strategies based on moderate-intensity exercise^18^.

## Results

All study participants completed the prescribed exercise protocol as scheduled, without reporting any injuries or discomfort. The average gait training speed for the APO group was 3.1 ± 0.4 km/h, as defined during the baseline (T0) assessment sessions.

### Gait efficiency

**Figure 1:**
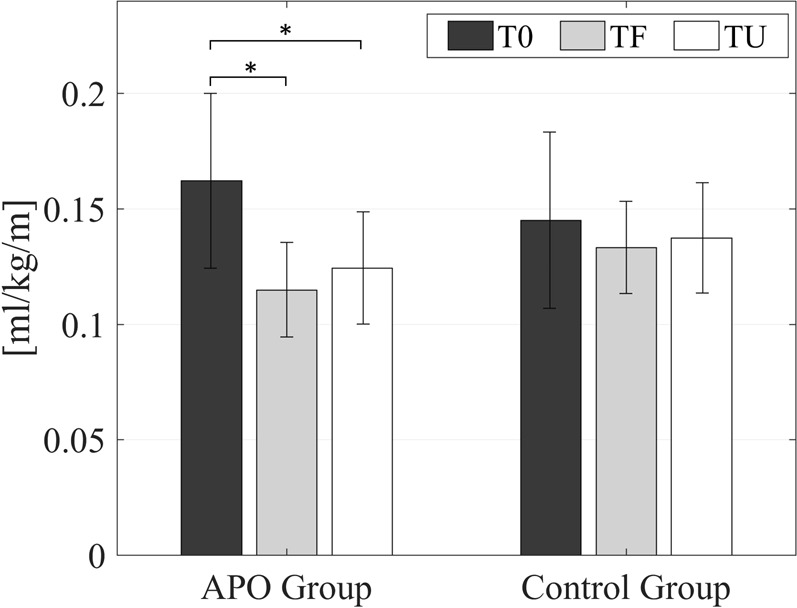
Average MCoT at T0, TF and TU for the APO and the control groups. Stars mark statistically significant differences.

Figure 1 displays the MCoT for each group at each of three time points: baseline (T0), post-training (TF – end of week 4), and at one-month follow-up (TU). No significant differences were found between the average MCoT of the APO group (0.162 ± 0.038 ml/kg/m) and the control group (0.145 ± 0.038 ml/kg/m) at baseline (p = 0.33). After training (TF), both groups exhibited a reduction in the MCoT with respect to T0 (APO: 0.115 ± 0.021 ml/kg/m; control: 0.133 ± 0.020 ml/kg/m), but only for the APO group was this reduction significant (APO: p < 0.01; control: p = 0.44). This reduction in MCoT remained significant for the APO group at the one-month follow-up (TU), though its magnitude was reduced (APO: 0.124 ± 0.024 ml/kg/m, p < 0.01; control: 0.137 ± 0.024 ml/kg/m, p = 0.44). Expressed in percentages, the APO training program resulted in a 26.6 ± 16.1% average reduction in MCoT at TF with respect T0, with a 20.2 ± 19.7% reduction maintained at TU (again relative to T0).

**Figure 2:**
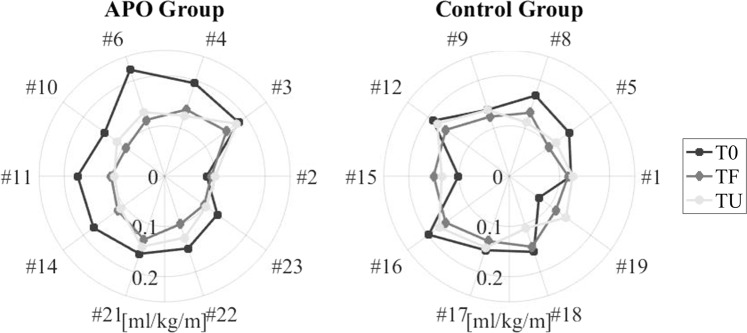
Individual Metabolic Cost of Transport results for the incremental tests repeated at T0, TF and TU. (IDs higher than 20 were assigned to the participants recruited after the exclusion of three subjects who did not meet the inclusion criteria).

These improvements in MCoT with APO-assisted training are further illustrated for individual subjects in Fig. 2. The visibly smaller areas encircled by the TF and TU lines relative to T0 for the APO group graph indicate a pronounced reduction in the MCoT with training. No such trend is visible for the control group.

### Robot-assisted training efficiency

**Figure 3:**
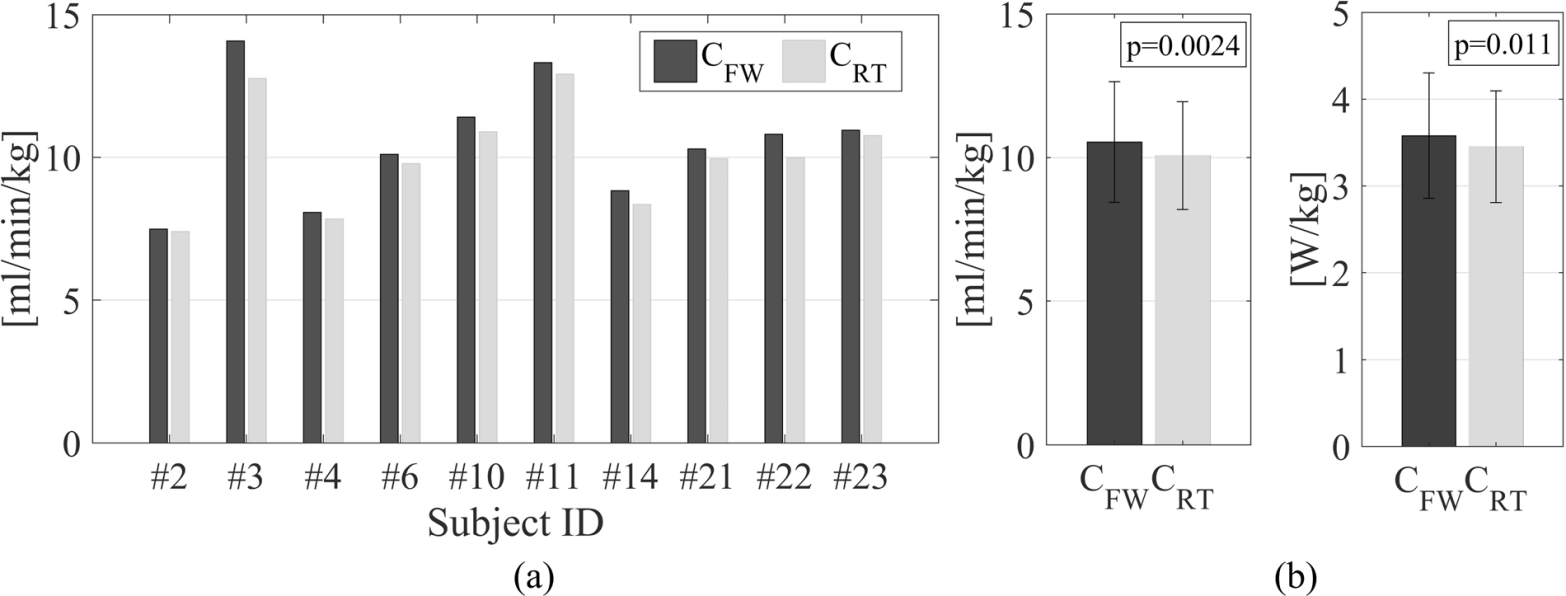
(**a**) Average individual Oxygen uptake rates for the last two minutes of treadmill 6-minute Walk Test without the APO (C_FW_) and under the APO-active training condition (C_RT_). (IDs higher than 20 were assigned to the participants recruited after the exclusion of three subjects who did not meet the inclusion criteria). (**b**) Average Oxygen uptake rate (left) and Metabolic Power (right) for the APO group under treadmill walking without the APO (C_FW_) and under the training condition (C_RT_).

Indirect calorimetry results of the APO group assessment (6 mWT) during the training period (TI) are shown in Fig. 3. All the subjects of the APO group demonstrated decreased oxygen consumption rates for the robotic training condition (C_RT_: 10.06 ± 1.88 ml/min/kg) compared to free walking without the APO (C_FW_: 10.54 ± 2.11 ml/min/kg). The metabolic power exhibited an analogous trend, decreasing from 3.58 ± 0.72 W/kg (C_FW_) to 3.45 ± 0.64 W/kg with APO assistance (C_RT_). Both the reduction in oxygen consumption and metabolic power under the training condition were statistically significant (p < 0.01 and p = 0.011, respectively). On average, the reduction in the oxygen uptake under the training condition was 4.24 ± 2.57%, while the corresponding reduction in metabolic power was 3.29 ± 2.88%. Under the training condition, the average steady-state metabolic consumption resulted in 3.87 ± 0.72 metabolic equivalents (METs), with heart rates of 59.2 ± 5.8% HR_max_.

**Figure 4:**
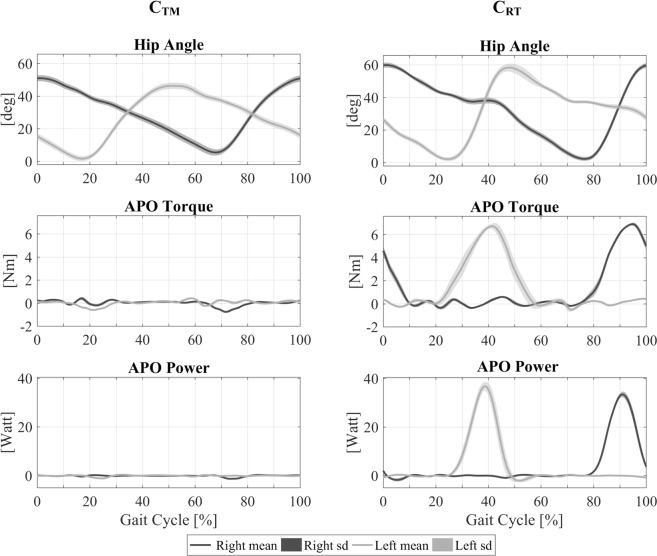
Right (dark grey) and left (light grey) angular, torque and power profiles of the APO under transparent mode (C_TM_) and training condition (C_RT_) for one sample subject.

The APO data from session TI are depicted in Fig. 4, which shows the average hip angle, torque, and power profiles recorded for a representative subject. From these profiles, it is evident that the APO flexion torque integrated into the gait cycle as intended, beginning slightly before hip flexion, reaching a peak during late flexion, and dropping to zero during the initial hip extension phase. Group results show that under the training condition, the APO transferred a flexor torque profile with mean peak value of 0.090 ± 0.008 N∙m/kg at 92.2 ± 2.3% of the stride phase, with 0% defined as the moment of maximum hip flexion. The mean positive power delivered by the APO to the hip joint during each stride was 0.049 ± 0.009 W/kg per leg, while positive peak values reached 0.441 ± 0.097 W/kg at 89.6 ± 2.6% of the stride phase. When receiving active torque assistance, subjects walked with an increased (19.4 ± 7.6%, p = 4.6e–5) range of hip motion relative to transparent (zero-torque) mode, as observed in the hip angle profiles in Fig. 4, which exhibit greater peak flexion for the training condition with respect to transparent mode. Moreover, the subjects showed higher (48.6 ± 17.4%, p = 2.0e–6) and delayed (9.2 ± 3.0%, p = 1.4e–5) peak angular velocities without significant variations in the stride period.

### Physical activity volumes

The results of the Physical Activity Scale for the Elderly (PASE) questionnaires taken at baseline and the actigraphies quantifying overall activity for the week before each assessment are summarized in Table 1. PASE scores of the two groups taken at baseline (T0) did not differ significantly (p = 0.69), nor did the average number of daily steps measured during the week prior to each assessment (p = 0.35 at T0; p = 0.39 at TF; p = 0.16 at TU). Nor were within-group variations in the number of daily steps between the three time points statistically significant (APO group, p = 0.82; Control group, p = 0.53).

**Table 1:** (a) Average number of steps walked each day during the three monitored weeks by each participant of the APO and control groups. (b) Median scores of the PASE questionnaires of the APO and control groups.

Daily steps (mean ± sd)	T0	TF	TU	PASE scores (median, iqr)	T0
APO	(9.89 ± 4.28)·10^3^	(11.2 ± 3.40)·10^3^	(9.77 ± 5.83)·10^3^	APO	(114,128)
Control	(12.1 ± 3.23)·10^3^	(12.7 ± 4.43)·10^3^	(11.5 ± 2.29)·10^3^	Control	(110,89)
**(a)**				**(b)**	

## Discussion

The aim of this study was to investigate the potential of a hip exoskeleton-assisted gait training program to increase unassisted gait efficiency in healthy elderly adults, relative to a traditional self-administered walking regimen, the most common clinical exercise prescription^19^. By incorporating an eccentric strength training element into moderate intensity aerobic exercise in accordance with established exercise guidelines for the elderly, the present study sought to maximize the training benefit-to-metabolic-cost ratio for the users.

The primary finding of this study was that gait efficiency improved significantly following APO-facilitated training but not for the control regimen of traditional free walking. In addition, walking with active assistance from the APO was found to incur a significantly lower metabolic cost than APO-free walking at the same speed, with respect to oxygen consumption and metabolic power.

To identify and understand the determinants of the observed metabolic improvements following robot-assisted training, several factors must be considered. First, though the authors recognize an incidental but notable difference in gender distribution between the APO and control groups resulting from randomization, it is unlikely that this difference contributed to the result, given that training effects on aerobic capabilities have been previously found to be gender-independent^9,41^.

A more likely influential factor on study results is the volume of exercise introduced by the two training programs. Notably, the number of average daily steps did not vary significantly between the pre-, during, and post-training periods of the study for either group, though marginal increases in the average numbers of daily steps during the training relative to baseline periods were observed for both groups. Though the slightly larger magnitude of this increase for the APO relative to control groups suggests that the observed difference in metabolic response between the groups may be partially attributable to a difference in total activity volume (a potential indicator of incomplete adherence by the control group), the lack of statistical significance of this observation renders firm conclusions unwarranted. Rather, as a matter of statistical sensitivity, the prescribed exercise regimens may not have constituted a significant increase in net weekly activity relative to the subjects’ usual lifestyles for either group, given the routine variation therein. It is further possible that some subjects in both groups offset their dedicated training sessions with concurrent decreases (either intentional or inadvertent) in their overall daily living activity. Thus, a larger study of greater statistical power would be required for decisive evaluation of training volume and exercise program adherence effects.

Nevertheless, the fact that the training period yielded significant improvements in gait efficiency without significantly higher step counts for the APO but not the control group suggests that the observed metabolic effects were at least partially due to the *quality* rather than the mere volume of the APO-facilitated exercise. This interpretation is further supported by the lack of significant cross-group differences in the total volume of activity between the APO and control groups during any of the three study phases, including training period.

In particular, the aerobic intensity of APO-facilitated training was likely a major contributor to increased gait efficiency following training. The average steady state heart-rate for the training condition was 59.2 ± 5.8% HR_max_, in line with the intended target of exercising at 60% HR_max_, while the average metabolic consumptions were of 3.87 ± 0.72 METs, thus ranking as a moderate-intensity physical activity^42^. On the other hand, walking at self-selected speeds usually falls among low-intensity activities, which elicit only mild heart rate increases, below the threshold for aerobic activity^18^ – and thus have a limited effect on cardio-pulmonary capacity in the elderly^43^. Critically, while the extent to which suspected differences in both training volume and intensity were enabled specifically by the APO’s provided torque assistance profile (relative to other factors such as motivational effects) cannot be precisely determined from the collected data, it may be concluded that APO-assisted training is conducive to training at both higher intensities and higher volumes than commonly achieved in clinical practice.

With regard to the potential strength training effects of APO training, the delivered torque profiles can be seen to have facilitated greater muscular elongation of hip extensors during the late hip flexion (swing) phase of gait, coinciding with the maximum flexor torque provided by the APO. The fact that the APO torque resulted in increased peak hip flexion without concurrent stride elongation (deduced from the constant stride period and treadmill speed) suggests a potential increase in muscular activation to effectively control the assistance provided by the APO – presumably including increased eccentric activation of hip extensors during late swing. Moreover, during their initial concentric contraction phase (onset of hip extension prior to heel strike), the APO provided a less powerful but still counter-acting flexion torque, thus requiring greater extensor activation.

Significantly, these torque profiles did not result in any additional overall effort for the user. To the contrary, the subjects showed an average 4.24 ± 2.57% reduction in oxygen uptake rates with the APO active, relative to the same condition without the APO. This result indicates that the positive power delivered by the APO during the initial flexion phase effectively compensated not only for the additional eccentric effort demanded during late swing and early stance, but also for the additional weight of the device. The net reduction in metabolic cost likely reflects both the balance of assistive to resistive power provided by the APO during the gait cycle, and also the increased metabolic efficiency of eccentric (vs. concentric) muscular contractions.

The APO’s performance in the present study compares favorably to other lower-limb exoskeletons that have been tested for their ability to reduce the metabolic effort of elderly subjects during locomotion. Galle *et al*. found a 4% non-significant reduction in net metabolic power when assisting ankle plantarflexion during the push-off phase, by transferring an average positive power of 0.11 W/kg each stride^37^— more than double the positive power delivered by the APO. Thus, the comparable reduction in energy expenditure achieved during APO-assisted gait (−3.29 ± 2.88% metabolic power, with −4.24 ± 2.57% oxygen consumption) represents a more efficient assistance strategy by the APO.

In a separate study using a hip exoskeleton, Lee *et al*. obtained a 7% reduction in the oxygen consumption rate^39^, by providing both hip extensor and flexor assistance with peak torque magnitudes comparable to the APO. Thus, the greater reduction in metabolic cost can be primarily ascribed to the higher total transferred power. Moreover, differences in torque phase between the two robotic systems may have contributed to the smaller metabolic reductions with the APO, which delivers a torque profile designed for eccentric training of the hip extensors, rather than optimized for lowering the real-time metabolic consumption. Indeed, moderate variations in exoskeleton torque timing can result in different metabolic efficiencies, with 60% of the gait cycle (GC) identified as the optimal timing for peak flexor torque in healthy young subjects^44,45^. Considering that the phase delay between peak hip flexion and heel strike ranges from 15 to 20%GC, the peak APO torque can be estimated to have been transferred between 72 and 77% of the gait cycle, corresponding to the phase of hip extensor activation commencing just prior to heel strike. By contrast, Lee *et al*.’s system reached the maximum amplitude at approximately 65%GC, closer to the optimal values for assistive torques.

The clinical significance of the present results may be appreciated with respect to previous studies that have reported improved gait efficiency of healthy elderly subjects following aerobic training^9,46^. These studies have shown increases up to 30% in $\dot{V}{O}_{2max}$ and/or gait efficiency, after programs of 3–6 months with 3 weekly training sessions of 30–60 minutes of training and intensities ranging from 50 to 85% of the maximal heart rate. In comparison, our training program was shorter, with exercise intensity (quantified by HR) in the lower half of this range. Therefore, the average 26.6 ± 16.1% decrease in MCoT following APO training observed in our study represents a remarkably strong outcome relative to the total burden of intervention. This improved benefit-to-cost ratio may be attributable in part to the eccentric strength training elements of our APO training program, given that strength training has been found to positively influence cardiorespiratory fitness^14^.

Of further note, the improvements in metabolic gait efficiency for subjects training with the exoskeleton remained statistically significant one month after the end of the training. The persistence of this metabolic effect beyond the training period is a clinically significant indicator of the training effect size of APO-mediated exercise, consistent with the hypothesis that APO-modulated assistance/resistance can amplify the effectiveness of exercise regimens in the elderly.

With respect to traditional exercise interventions, current literature provides sparse reference regarding the follow-up measurement of metabolic training effects of exercise interventions in older adults, with the majority of the studies limiting long-term post-training assessments to the evaluation of training adherence rather than of the retained metabolic effects^47^. However, Di Lorenzo and colleagues have reported that the physiological improvements induced by a 12-week aerobic training program in adults were still significant at 1-year follow-up^48^, and similar trends were reported for patients with chronic obstructive pulmonary disease^49^ and arthritis^50^. As in our study, in all cases the subjects informally reported that they continued to perform physical activity to an equal or lesser extent following the training intervention.

Another significant aspect of the present study was its demonstration of the clinical feasibility and suitability of APO-based training to elderly individuals. Specifically, the APO’s demonstrated ability to lower the energetic demand of a given volume of training may promote better engagement and long-term adherence to training prescriptions, by reducing the perceived burden of training while potentially enhancing the metabolic benefits. These capabilities may prove valuable for elderly users across the health and physical ability spectrum. For active elderly users, exoskeletons like the APO may be used to implement progressive training paradigms with gradually increasing intensities. For frail users with low residual mobility, such exoskeletons may directly restore mobility, by substantially lowering the effort and/or increasing the capacity for locomotion using purely assistive strategies.

The main limitation of the present study was its inability to isolate and quantify the role of the APO torque modulation strategy *per se* as a determinant of the observed metabolic training effects, relative to the concurrent factors of exercise volume and intensity. Moreover, with regard to activity volumes and intensities beyond the APO training sessions (including baseline and follow-up phases), the actigraphy data were not able to specifically address activity intensity, nor to distinguish dedicated training sessions from overall activity. Given the equivalence of instructions to subjects during non-training periods and the lack of significant differences in daily step counts between phases (intra-group) and between groups (intra-phase), it is reasonable to attribute the observed difference in outcome primarily to the differences between the APO and control training regimens, rather than to any difference in non-training activities.

In particular, the observed improvements in gait efficiency following APO training are likely attributable in part to the higher aerobic training intensity facilitated by the APO with respect to natural walking, as well as to the additional load (mass) of the robot. Though the present study was not designed to parse the relative contributions of these factors, it remains clear that the APO can facilitate walking at higher volumes, speeds, and/or aerobic intensities than may otherwise be comfortably achieved by unassisted overground walking, the current standard of care. This difference would be expected to be more pronounced for more frail individuals, who represent the highest overall health risk and thus the highest clinical need.

Another potential confound in the present study was the difference between treadmill and overground walking between the APO and control groups. While treadmill walking has itself been found to incur an additional metabolic cost relative to overground walking at the same speed in the elderly^51^, this effect has been found to diminish significantly with a familiarization period of just 15 minutes^52^, suggesting that the effect may be a transient artifact of short-term adaptation to treadmill walking for previously-unfamiliar individuals. Thus, the treadmill walking familiarization period afforded to both groups immediately prior to the treadmill-based MCoT assessments at baseline – and only to the control group for subsequent assessments – may be expected to have mitigated the potential contribution of group-dependent treadmill training effects to the result. Given the established similarity between treadmill and overground gait biomechanics^53^, it is thus unlikely that the improved MCoT response in the APO relative to control group was a product of intrinsic differences between the two forms of training. However, it remains possible that the differences in the structure and supervision of the clinical versus home environments could have been a contributing factor, via differential effects on subject motivation and protocol adherence.

In conclusion, this study demonstrated the feasibility and potential benefits of training with an exoskeletal robotic active pelvis orthosis (APO) to elicit positive effects on cardiorespiratory fitness in healthy elderly users. For active elderly individuals, the APO-assisted training strategy was more effective in increasing metabolic gait efficiency than the clinical standard of self-administered walking practice. Significantly, to the authors’ knowledge, this study was the first of its kind to demonstrate a positive training effect of robotically assisted gait training on the metabolic gait efficiency in healthy elderly adults. The preservation of the observed training effect with only mild attenuation over a one-month follow-up period provides further support for the use of exoskeletons as gait training aides in the elderly. Moreover, metabolic study data demonstrate the suitability of the proposed APO training strategy with respect to widely recognized clinical guidelines for safe, sustainable, and effective exercise strategies to promote active aging in the elderly.

The present study thus constitutes a promising proof of feasibility that paves the way for the further investigations of exoskeletons as enabling technologies for reversing sedentary lifestyles and promoting healthy, active aging in the elderly. With this objective, future studies will more rigorously evaluate the effectiveness of robot-assisted training compared to traditional exercise programs better controlled and quantified with respect to training type, volume, and intensity. These investigations will seek to elucidate the specific physiological mechanisms, benefits, and potential limitations of robot-assisted training, by distinguishing the effects of strategic assistance-resistance modulation from the benefits derived from the overall metabolic load of exercise. In addition to metabolic outcomes, such studies should directly evaluate adaptive modifications in muscle activation patterns and gait biomechanics in response to sustained exoskeleton-based training. With the resulting understanding, optimized torque modulation strategies may be developed to meet a range of clinical objectives for a corresponding range of users.

## Materials and Methods

### Participants

Twenty healthy elderly volunteers were recruited for the study. Suitable candidates were identified as moderately active individuals over age 65, in good health. Age over 85, the presence of cognitive impairments (Mini Mental State Examination < 21), marked anxiety or depression (Geriatric Depression Scale-GDS and Patient health Questionnaire-PHQ-9^54,55^) or any disease either reducing the walking abilities or counterindicated for aerobic physical activity, constituted exclusion criteria^56^. The participants were randomly assigned to one of two groups: the ten participants in the experimental APO group (age 70 ± 5 years; 2 female/8 male; BMI 28.4 ± 2.9 kg/m^2^) followed a robot-assisted training program, while the ten participants in the control group (age 70 ± 4 years; 7 female/3 male; BMI 28.7 ± 4.5 kg/m^2^), performed self-paced overground walking.

The experiment was conducted at the Fondazione Toscana Gabriele Monasterio (Pisa, Italy), in accordance with institutional regulations, and under the approval of the local ethics committee (IRB of Azienda Ospedaliero-Universitaria Pisana, Study n. 1202, Prot. 67053). Written informed consent was provided by each participant.

### The Active Pelvis Orthosis (APO)

The APO is a powered robotic exoskeleton that actively modulates hip flexion and extension movements during locomotion. The APO system used for this study was based on the same mechatronic architecture as a previously reported prototype^57^, with additional design optimizations for full portability and weight reduction to 6.5 kg.

The APO is designed to gently power hip movements by providing smooth torque profiles at the pelvis level, adapting to natural gait variations. The desired torque pattern is computed through high-level algorithms relying on accurate gait phase recognition for synchronization of the assistive action with the motion of the user. Precise gait-phase estimation is obtained by continuously tracking the hip kinematics through the embedded joint angle sensors and using maximum hip flexion events for cyclic phase-reset^58^.

The relatively low output impedance of the APO combined with reliable real-time knowledge of the gait phase allows for modulation of the torque in order to obtain purely assistive as well as partially resistive torque profiles. For this study, the adopted torque strategy was specifically conceived to (i) assist the final phase of hip flexor action and (ii) induce augmented eccentric contraction of hip extensors, to maximize the training effects without introducing an excessive burden for the user.

**Figure 5:**
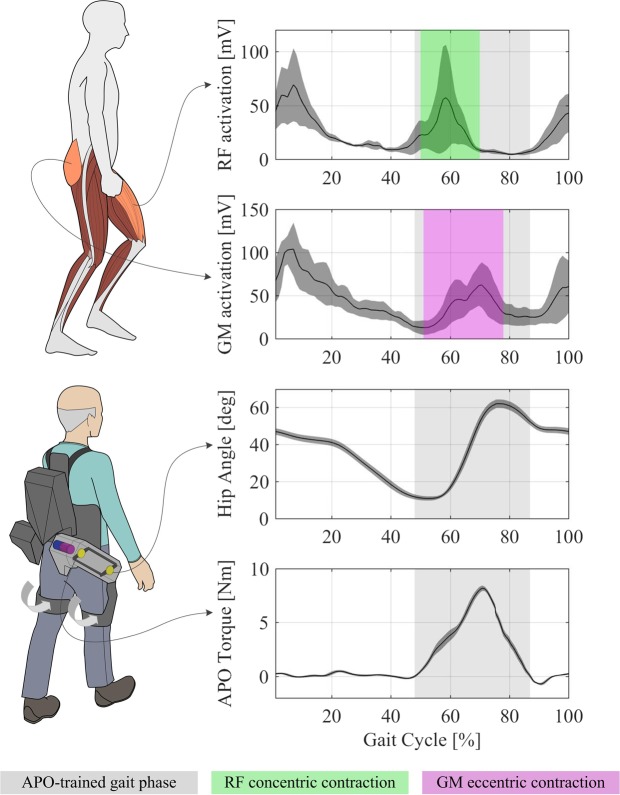
Torque strategy of the APO. Based on literature data^59^ of hip muscular activation, flexor torque profiles were phased in order to assist the *Rectus Femoris* (hip flexor) contraction and resist the activation of the *Gluteus Medius* (hip extensor).

Torque profiles were shaped in accordance with the muscle activation profiles observed in literature during walking^59^. These profiles are characterized by activation of the Rectus Femoris – a primary hip flexor – until 70% of the gait cycle; at that time, Gluteus Medius – a primary hip extensor – is maximally activated during the swing phase^59^ (Fig. 5). Hence, the torque profiles provided by the APO were modeled as positive (i.e. flexion torque) half-period sinusoidal curves with peak amplitude reached during late flexion, to concurrently assist hip flexors and resist hip extensors, thereby augmenting the negative work of the extensors (Fig. 5). In addition, the residual flexion torque provided during the early stance phase may elicit further hip extensor activation in their concentric phase. Maximum torque values were set around BM/10 Nm/kg (BM = body mass), while torque duration was fixed to 40% of the stride period, as torques with shorter periods or higher peak values were generally perceived as uncomfortable or difficult to manage by the users.

### Study design

**Figure 6:**
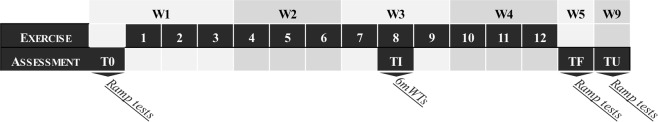
Schedule of the exercise and assessment sessions throughout the experimentation weeks.

The study protocol lasted for a period of 2 months per subject, consisting of a four-week exercise intervention, followed by a one-month follow-up period. All subjects performed both *exercise* and *assessment* sessions according to the timetable reported in Fig. 6.

*Exercise* sessions required the subjects in both groups to complete 3 weekly 1-hour sessions of physical activity, in different forms. To represent the current standard of clinical care, the control group was assigned a home exercise program of self-paced overground walking, to be conducted in an unstructured environment (presumably outdoors – as available to each subject). Meanwhile, members of the APO group performed treadmill-based walking sessions with the APO in a clinical laboratory environment, in order to maximize subject safety during this initial feasibility study, by providing handrails and immediate supervision to protect against potential falls during the initially unfamiliar task of APO-modulated walking. Further, the controlled, constant gait velocity enabled by treadmill walking allowed the APO to provide assistive torque profiles of maximum regularity, thus promoting both subject safety and data uniformity. Based on the established similarity between treadmill and overground gait biomechanics^53^, the two forms of walking were deemed adequately comparable for the present study.

The exercise program of the APO group was designed to incorporate both aerobic and strength training features: it consisted of treadmill walking with the APO providing gait phase-specific hip flexor assistance and extensor resistance according to the torque strategy described above, at a treadmill speed selected to result in a submaximal-intensity aerobic exercise. Individual fine-tuning of torque amplitude was accomplished during the first session and maintained constant throughout the study.

Three *assessment* sessions were conducted to assess the subjects’ metabolic gait efficiency: one at baseline (T0), prior to the beginning of the training period, a second at its conclusion (TF), and a one-month follow-up (TU). During these sessions, the metabolic gait efficiency of the participants was assessed by means of an incremental treadmill-based ramp exercise test^60^: subjects were required to walk on a treadmill at increasing speeds, starting from 2.0 km/h and gradually incrementing by 0.5 km/h every two minutes. The test started after a baseline (resting) recording period and continued until either (i) reaching a heart rate equal to 75% of the maximal heart rate (defined as HR_max_ = (210-Age∙0.65) ± 10^61^) – or (ii) approaching the anaerobic threshold, as determined by the respiratory exchange ratio (RER) of carbon dioxide output to oxygen uptake rates exceeding 0.95^62,63^. All subjects from both groups performed the ramp test without the APO.

Data from the ramp tests at T0 were also used to set training speeds for the subjects of the APO group. Treadmill training speeds were chosen corresponding to an HR equal to 60–65% of HR_max_, to achieve a target of 60% HR_max_ during APO-assisted training, in line with clinical recommendations for moderate-intensity exercise^26^. To ensure stable metabolic measurements and minimize the potential for data artifacts due to variations in treadmill walking familiarity, all subjects in both groups were provided a treadmill familiarization period of up to 10 minutes prior to the baseline (T0) MCoT assessment, until they reported and demonstrated a fully level of comfort. Subjects in the control group repeated this same familiarization procedure at the beginning of subsequent treadmill-based assessment sessions (TF and TU) as well, while those in the APO group were assumed to be adequately familiarized to treadmill walking from their training sessions.

In addition to the evaluations at T0, TF, and TU, the APO group participated in a fourth assessment (TI), to evaluate the efficiency of robot-assisted locomotion. This assessment was conducted at the eighth training session, when subjects were assumed to be fully familiarized with the provided torque profiles and to have established a stable pattern of motor adaptation. During this session, the energetic demand of walking was evaluated in two treadmill-based 6-minute Walking Tests (6 mWT): one with the APO active, under the training condition (C_RT_), and the other without the APO (C_FW_), representing to the control condition.

The two 6 mWTs were conducted at the same walking speed, and the order of the conditions was randomized across subjects. At the beginning of the test, baseline oxygen consumptions were recorded with the subjects standing still. After the two 6 mWTs, an additional 5 minutes of treadmill walking were recorded with the subjects wearing the APO in “transparent mode” (C_TM_), to serve as baseline reference for the torque and kinematics data recorded by the APO under the C_RT_. In C_TM_, the APO employs an active torque-control system to negate the robot’s passive resistance and maintain zero externally applied joint torque, so as to provide neither assistance nor hindrance to the user’s movements.

### Data collection and analysis

All primary outcome data were collected on the assessment session days, at T0, TF, TU, and TI. During the assessment tests, i.e. the incremental ramp tests and the 6 mWTs, each subject’s oxygen uptake (VO_2_, [ml/min/kg]), carbon dioxide output (VCO_2_, [ml/min/kg]), and heart rate (HR, [min^−1^]) were monitored using the Oxycon Mobile device (Jaeger, Germany). Spatiotemporal data including treadmill speed profiles, training times, and distances walked were recorded for all treadmill-based assessment and training sessions.

The acquired data were analyzed offline in MATLAB (MathWorks Inc., Natick, Massachussetts, USA), to assess changes in gait efficiency related to robot-assisted training. The data collected during the ramp tests were used to compute the Metabolic Cost of Transport (MCoT, [ml/kg/m]) at T0, TF, and TU, as an indicator of the gait efficiency and cardio-pulmonary fitness of the subjects throughout the study. The MCoT was obtained according to the following formula:

$$Metabolic\,Cost\,of\,Transport=\frac{{\int }_{{t}_{start}}^{{t}_{stop\_{v}_{f\_{\min }}}}({\dot{V}}_{{O}_{2}}-{V}_{{O}_{2BL}}^{\cdot })dt}{distance}$$

${\dot{V}}_{{O}_{2}}$ [ml/s/kg]: filtered Oxygen uptake rate

${V}_{{O}_{2BL}}^{\cdot }$ [ml/s/kg]: average baseline Oxygen uptake rate recorded during the last minute of standing still

*t*_*start*_ [s]: walking start time

*t*_*stop_vf*___*min*_ [s]: test end time for the minimum final velocity, among T0, TF and TU

*distance* [m]: distance covered from *t*_*start*_ to *t*_*stop_vf*___*min*_

All statistical analyses were performed with a significance level of α = 0.05. Changes in MCoT over the course of the training and follow-up periods were evaluated within each group by applying a one-way repeated measures ANOVA, with normality of sample distributions verified by Lilliefors test. For groups found to have significant differences in MCoT between time points (collectively), paired 2-tailed t-tests were performed to identify significant differences between specific time points, by comparing the MCoT at TF and TU (respectively) with the baseline values obtained at T0. To verify comparable levels of baseline fitness between groups, an independent samples t-test was applied to the sets of MCoT values at T0 between the two groups.

In addition to MCoT, indirect calorimetry data collected during the 6 mWTs (APO group, at TI) were used to estimate the steady-state energetic expenditure. To this end, VO_2_, VCO_2_, and HR raw signals were filtered and averaged over the last two minutes of each condition, when a plateau in the consumption rates had been reached. The average steady-state metabolic power was computed according to the Brockway equation^64^. To classify the intensity of activity under the training condition, the adapted resting metabolic rate of 2.6 ml/kg/min found for older adults by^65^ was adopted for conversion of VO_2_ levels to METs. Statistical significance of the variations in the energetic consumptions between the C_RT_ and C_FW_ conditions was assessed using 2-tailed independent t-test.

During the 6mWT under C_RT_ and the following 5 minutes under TM, the APO joint angular and torque profiles were acquired by the onboard sensors at 100 Hz. These signals were segmented into individual strides using the maximum hip flexion angle, which was used as the 0% gait phase reference event. Hip angle profiles were time-normalized and interpolated to a scale of 0–100% of stride period, then averaged to obtain the mean kinematic profiles under the recorded conditions. The orthosis power was computed by multiplying the hip joint angular velocity by the APO-exerted torque. Stride mean positive power (representing net work done by the APO per stride) was obtained by dividing the integrated positive power profile over each stride by its duration, and then averaging this value across all strides.

To verify the equivalency of activity levels between the two groups (quantified by daily total number of steps), all subjects were provided and instructed to wear wrist-based activity monitors (Fitbit Flex 2TM, USA) throughout the week prior to each assessment (T0, TF, and TU), including during training sessions – thus quantifying subject activity during the baseline, training, and follow-periods, respectively. Actigraphy data from each monitoring period were downloaded at the corresponding assessment session, and were taken as representative of overall physical activity levels throughout each period. As an additional descriptive measure for comparison between groups, all subjects completed the Physical Activity Scale for the Elderly (PASE) questionnaire^66^, a self-reported questionnaire of perceived activity level, pertaining to their usual activity levels prior to the study. Subjects were not instructed to modify their activity regimens or behavior from their usual routines during either the baseline or follow-up periods.

Potentially significant differences in PASE and actigraphy measures between the APO and control groups were assessed by Mann-Whitney U-tests, while variations occurring over time within each group were evaluated separately using the Kruskal-Wallis non parametric ANOVA test. This test was chosen over the Friedman non-parametric ANOVA for repeated measures due to the presence of missing samples in the wearable actigraphy data resulting from imperfect subject adherence, which precluded the use of paired-measures test.

## Data Availability

The datasets generated during and/or analysed during the current study are available from the corresponding author on reasonable request.
